# A Meta-Analysis of the Risk Factors of Persistent Pulmonary Hypertension in Newborns

**DOI:** 10.3389/fped.2021.659137

**Published:** 2021-10-29

**Authors:** Ran Zhou, You-Ning Zheng, Xin-Ying Zhang, Ya-Ying Cheng

**Affiliations:** Department of Pediatrics, HeBei General Hospital, Shijiazhuang, China

**Keywords:** newborn, pulmonary hypertension, risk factors, case control study, meta-analysis

## Abstract

**Objective:** To determine the risk factors of persistent pulmonary hypertension of the newborn using a meta-analysis method and provide a reference for its clinical prevention and treatment.

**Methods:** A meta-analysis was performed by searching the PubMed, Embase, Cochrane Library, China Biology Medicine Disc, Wanfang, and Chinese VIP journal databases, as well as the China National Knowledge Infrastructure.

**Results:** A total of 22 references were included in the meta-analysis; the cumulative medical records comprised 7,937 cases, and 2,613,072 control cases were included. A total of 12 related risk factors were included (7 were associated with pregnant women and 5 were associated with newborns).

**Conclusion:** Among the 12 associated risk factors included, the three most important and their combined odds ratio values and 95% CI were as follows: (1) pregnant women smoking, 4.85 (1.98–11.9) during pregnancy; (2) gestational weeks <37, 4.34 (1.64–11.5); (3) perinatal asphyxia, 3.9 (2.87–5.31).

## Introduction

Persistent pulmonary hypertension of the newborn (PPHN) refers to the persistent increase in pulmonary vascular resistance after birth, resulting in pulmonary artery pressure exceeding the systemic arterial pressure, the right-to-left shunting of blood at the atrial and/or ductal level, severe hypoxia, and other clinical symptoms (1–3). The disease has a high mortality and disability rate in neonatal intensive care units and a high incidence of morbidity in later life. The incidence of PPHN reported in the USA is ~1–5%. However, accurate epidemiological data in this regard are lacking in China (4).

At present, PPHN is not considered a single disease but a clinical syndrome caused by many factors that may be correlated to genetic and environmental factors (5). However, there is no unified understanding of the risk factors and pathogenesis of PPHN. In addition, the early diagnosis of PPHN will be of great significance in terms of follow-up treatment; however, there is currently no method-specific or sufficiently sensitive diagnostic for doing so. This study investigated the risk factors of PPHN by conducting a meta-analysis.

## Materials and Methods

### Literature Retrieval Strategy

This study searched the PubMed, Embase, Cochrane Library, and other foreign electronic databases for relevant articles. Chinese electronic databases, such as China Biology Medicine Disc, Wanfang, Weipu, and China National Knowledge Infrastructure, were also queried. In English, “newborns,” “risk factors,” “persistent fetal circulation syndrome,” and “randomized controlled trial” were employed as subject headings, and a combination of subject and free-text terms were used to conduct searches. The Chinese search words were as follows: “新生儿” (newborns), “持续性肺动脉高压” (persistent pulmonary hypertension), and “危险因素” (risk factors).

### Literature Selection and Literature Quality Evaluation

#### Literature Selection

The inclusion criteria related to database journals were as follows: case–control studies on the risk factors of developing PPHN; all cases were confirmed PPHN; the sample size was provided; odds ratio (OR) value and a 95% CI were provided or data in the text could be converted into OR value and a 95% CI; the relevant journal was written in Chinese or English.

The exclusion criteria were as follows: the journal articles were reviews or a meta-analysis; articles related to individual cases or that lacked control groups; duplicate documents; articles with incomplete data or where the full text was unavailable.

Two independent investigators reviewed each of the studies and determined if they should be included based on the inclusion and exclusion criteria, and disagreements between them were resolved through a third party or by both parties.

#### Quality Evaluation of the References

The Newcastle–Ottawa scale was used to evaluate the methodological quality of the included references. The results are shown in Table 1.

**Table 1:** The basic situation of these literatures.

**No**.	**First author**	**Published time**	**Place**	**Number in the observation groups**	**Number in the control groups**	**NOS score**
1	Reece (6)	1987	US	37	150	7
2	Van Marter (7)	1996	US	138	298	9
3	Bearer (8)	1997	US	31	39	6
4	Muraskas (9)	2001	US	15	36	7
5	Chambers (10)	2006	US	337	836	7
6	Kallen (11)	2008	Sweden	2,006	830,818	9
7	Araujo (12)	2008	Brazil	43	130	7
8	Andrade (13)	2009	US	1,104	1,104	8
9	Cai (14)	2010	China	45	82	7
10	Li (13)	2011	China	57	114	7
11	Li (15)	2011	China	80	80	8
12	Wilson (9)	2011	US	20	120	8
13	Liang (16)	2011	China	40	103	9
14	Delaney (17)	2012	US	337	846	8
15	Yang (18)	2013	China	57	114	7
16	Shen (19)	2013	China	41	41	7
17	Qi (20)	2014	China	92	92	8
18	Wang (21)	2014	China	57	63	7
19	Lee (22)	2014	Korea	28	32	8
20	Li (23)	2015	China	50	50	8
21	Du (24)	2016	China	45	45	7
22	Steurer (25)	2017	US	3,277	2E + 06	8

### Data Acquisition and Processing

The “double independent extraction method” was performed. The references were extracted by two evaluators according to the data collection table (Table 1), and controversial data were explored using a third investigator or jointly by both investigators to determine whether they should be included. Dichotomous data considered the OR as the effect size; based on whether the same measure was adopted for the continuous variable, the mean difference or the standardized mean difference was used as the effect size. The Revman 5.3 software (https://training.cochrane.org/online-learning/core-software-cochrane-reviews/revman/revman-5-download) program was used to merge effect sizes and to analyze the heterogeneity of the included data. If no heterogeneity was detected (p > 0.1 and *I*^2^ < 30%), the fixed-effects model was directly applied. If heterogeneity was detected (*p* < 0.1 or *I*^2^ > 30%), the random-effects model was used. The Beggar method was adopted to analyze the publication bias of the included studies.

## Results

### Literature Retrieval Result

**Figure 1:**
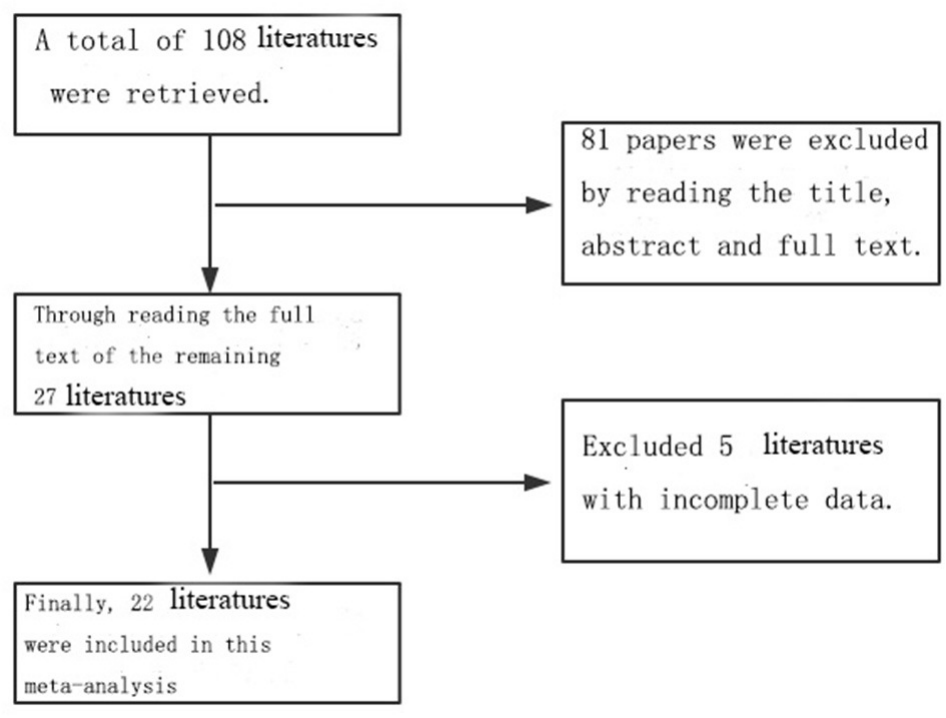
The literature screening flow chart.

A total of 108 related references were retrieved according to the adopted retrieval strategy; 27 articles were initially included after reading the title, abstract, and full text of the references, and five references with incomplete data were excluded. A total of 22 articles were finally included in this study, all of which represented case–control studies. In total, there were 7,937 case records and 2,613,072 control cases. The process chart for the inclusion of articles is presented in Figure 1, and the basic background of these references is presented in Table 1.

### The Combined Literature and Heterogeneity Test Results

The included references were combined according to the 12 potential risk factors of PPHN. The applied effect model was selected based on the heterogeneity, in which p >0.1 and *I*^2^ < 30% indicated no heterogeneity, 0.01 < *p* < 0.1 or 30% < *I*^2^ < 60% indicated middle heterogeneity, and 0 < *p* < 0.01 or 60% < *I*^2^ indicated larger heterogeneity. The fixed-effects model was selected for cases with no heterogeneity, while the random-effects model was selected for cases with heterogeneity (Table 2).

**Table 2:** The combined literature and heterogeneity test results.

**Risk factors**	**The number of literatures**	**References**	***P***	***I* ^**2**^**	**Heterogeneity**	**Effect model**
BMI >27	6	(10, 13, 20, 22–24)	*0.42*	*13.30%*	No	Fixed
Age >30	6	(10, 13, 20, 22–24)	*0.34*	*15.20%*	No	Fixed
Pregnancy-induced hypertension syndrome	8	(9, 13, 16, 18–20, 23, 25)	*0.07*	*49.50%*	Middle	Random
Gestational diabetes mellitus	9	(8, 10, 13, 16, 18–20, 24, 26)	*0.03*	*52.80%*	Middle	Random
Smoking	8	(7, 10, 13, 17, 22, 24–26)	*0.14*	*29.20%*	No	Fixed
Taking drugs	10	(7, 9–11, 13, 14, 17, 24–26)	*0.5*	*10.60%*	No	Fixed
Unipara	5	(9, 13, 17, 22, 24)	*0.29*	*21.60%*	No	Fixed
Newborn gender	11	(7–10, 13, 15, 18, 19, 24–26)	* <0.01*	*81.80%*	Larger	Random
Neonatal asphyxia	11	(8, 9, 15–21, 23, 25, 26)	*0.07*	*33.80%*	Middle	Random
Gestational weeks >42	6	(9, 15, 20, 22, 23, 26)	*0.36*	*22.20%*	No	Fixed
Cesarean delivery	13	(6, 9, 13, 15, 18–21, 23–26)	*0.15*	*10.40%*	No	Fixed
Gestational weeks <37	8	(13, 15, 16, 18, 19, 22, 25, 26)	*0.25*	*9.10%*	No	Fixed

### Combined Risk Factors

#### The Risk Factors Related to Pregnant Women

In this study, seven major risk factors were found to be associated with pregnant women, i.e., age, body mass index, unipara, pregnancy-induced hypertension syndrome, gestational diabetes mellitus, smoking, and the use of selected prescription drugs. A history of smoking included pre-pregnancy and mid-pregnancy smoking, and prescription drug use included steroid hormones, selective serotonin reuptake inhibitors (SSRIs), and other antidepressants. The OR values of the aforementioned risk factors were combined, and the publication bias and sensitivity were analyzed and calculated. The results are presented in Table 3.

**Table 3:** Risk factors present in pregnant women.

**Risk factors**	**Combined**	**Effect**	**Combined**	**95% CI**		**Publication**	**Sensitivity**
	**quantity**	**model**	**OR value**			**bias**	**analysis**
				**Lower limit**	**Upper limit**		
BMI >27	6	Random	1.32	0.92	1.89	No	Consistent
Age >30	6	Random	2.8	0.36	2.93	No	Consistent
PIH	8	Random	2.42	0.73	8.05	No	Consistent
Gestational diabetes mellitus	9	Random	3.61	2.02	6.45	No	Consistent
Pre-pregnancy and mid-pregnancy smoking	8	Random	4.85	1.98	11.9	No	Consistent
Taking drugs	10	Random	2.39	1.25	4.57	No	Consistent
Unipara	5	Random	1.16	1.09	1.23	No	Consistent

The combined OR value of the seven risk factors was >1. The most important risk factors were smoking, both pre- and mid-pregnancy (pre-pregnancy), and pregnant women with diabetes. The combined OR and 95% CI values were 4.85 (1.98–11.90) and 3.61 (2.02–6.45), respectively. Publication bias and sensitivity analyses were carried out for the seven combined risk factors, which were found to be free of publication bias and were consistent in terms of sensitivity.

#### Risk Factors Associated With Newborns

Five risk factors associated with newborns were included in the present study, i.e., male gender, perinatal asphyxia, gestational weeks <37, gestational weeks >42, and cesarean delivery. The most important risk factor was gestational weeks <37, and the combined OR and 95% CI value was 4.34 (1.64–11.50). Publication bias and sensitivity analyses were carried out for the five combined risk factors, which were found to be free of publication bias and consistent in terms of sensitivity (Table 4).

**Table 4:** Risk factors present in newborns.

**Risk factors**	**Combined**	**Effect**	**Combined**	**95% CI**		**Publication**	**Sensitivity**
	**quantity**	**model**	**OR value**			**bias**	**analysis**
				**Lower limit**	**Upper limit**		
Baby boy	11	Random	1.84	1.28	2.63	No	Consistent
Neonatal asphyxia	11	Random	3.9	2.87	5.31	No	Consistent
Gestational weeks <37	8	Random	4.34	1.64	11.5	No	Consistent
Gestational weeks >42	6	Random	1.75	0.97	3.18	No	Consistent
Cesarean delivery	13	Random	2.06	1.34	3.15	No	Consistent

## Discussion

Persistent pulmonary hypertension of the newborn remains one of the main causes of postnatal death. In addition, lacking data for improving awareness of the factors that influence the early diagnosis and prevention of PPHN increases the risk of developing the condition. At present, the risk factors for PPHN are not fully understood and several of these risks remain controversial. Clarifying the risk factors of PPHN is not only of significance to the prevention of PPHN but can also provide awareness for early clinical detection and diagnosis of the condition.

Among the 12 risk factors in this study, 8 associated with an OR >2 were pre-pregnancy and mid-pregnancy smoking, gestational weeks <37, perinatal asphyxia, pregnant women with diabetes, the age of pregnant women being >30, pregnant women with pregnancy-induced hypertension (PIH), and cesarean delivery.

The mechanism of PPHN caused by pregnant women who smoked may be correlated to the presence of nicotine in tobacco. According to a study conducted by Bearer et al. (8), cotinine and the metabolites of nicotine in the human body can rapidly accumulate in the fetus through entry *via* the placenta. In addition, a fetal liver does not have the ability to metabolize cotinine; hence, the half-life of cotinine in the uterus is prolonged. In addition, the decrease in blood oxygen content caused by smoking pregnant women is also a risk factor for PPHN. According to a study conducted by Van Marter et al. (25), low blood oxygen content in pregnant women can cause the muscle layers of neonatal arterioles to overdevelop before birth. Under hypoxia stimulation, pulmonary arteriole spasm leads to a continuous increase in pulmonary artery pressure and an increase in pulmonary artery resistance. Factors such as a total gestational period of ≤ 37 weeks, perinatal asphyxia, pregnant women with diabetes, pregnant women aged above 30, and pregnant women with PIH may affect the blood supply of the placenta to the fetus and lower the fetal blood oxygen concentration. Furthermore, fetal lung development is affected by chronic hypoxia of the fetus in the uterus (7).

Premature infants are prone to neonatal respiratory distress syndrome. Due to the insufficient secretion of alveolar surfactant, the development of pulmonary vessels and parenchymal tissue is inferior to that of full-term infants; accordingly, the ventilation function of premature infants is weak, and pulmonary artery spasm and other phenomena can easily occur, leading to the increased or even continued increase of pulmonary artery pressure and other risks. Among the risk factors of PPHN in late pregnancy, existing research (27) included placental abscess, preeclampsia, intrauterine growth restriction, and other diseases; the risk factors selected in this study included preeclampsia, and pregnancy combined with diabetes.

The full-term newborns of diabetic mothers tend to be heavier. Some studies (28) reported that heavier fetuses also had a higher incidence of PPHN because heavier children consume more oxygen, which further increases the incidence of pulmonary hypertension. Preeclampsia can cause fetal chronic intrauterine hypoxia; furthermore, intrauterine chronic hypoxia and perinatal asphyxia can lead to neonatal hypoxia and acidosis in the body, pulmonary arteriole endothelial damage, and artery deformation and mechanization; this can, in turn, lead to increased pulmonary artery pressure. As a result, hypoxia can become aggravated, and the pulmonary arteriole can continue to spasm, leading to the formation of PPHN.

An increasing amount of evidence suggests that the type of newborn delivery is also a risk factor for developing PPHN. A case–control study based on 9,452 infants in the USA showed that the risk of PPHN among cesarean section delivery was five times higher than in cesarean section with rates of 0.5% (42/8,388) and 0.09% (1/1,064), respectively (13). The increased risk of PPHN during a cesarean section may be (29) related to prostaglandins; this type of delivery can reduce the level of prostaglandins in newborns, which acts as the main vasoactive factor for infants to quickly adapt to the uterine environment after birth (7). In addition, a cesarean section does not include natural birth canal extrusion; as a result, residual amniotic fluid in the interstitium of the newborn's lungs will impact the amount and function of lung surface-active substances, thereby affecting the contraction and diastole of the alveoli.

In addition to the previously noted risk factors, the incidence of PPHN in male infants is higher than in female infants; the combined OR and 95% CI was 1.84 (1.28–2.63). The analysis presented herein is consistent with the existing literature in this regard, which may be related to the lower maturity of male lung development in the neonatal period, making male infants more prone to suffering from PPHN compared with female infants. Steroid use during pregnancy and SSRI-type antidepressants are also risk factors for PPHN, but the specific mechanism(s) involved remains unclear. Current evidence suggests that the use of SSRIs during late pregnancy increases the risk of newborns developing PPHN (30–32).

In summary, PPHN is a disease caused by a combination of multiple factors. Reducing adverse factors in the perinatal period and actively intervening to correct metabolic disorders after birth, as well as avoiding excessive intrathoracic pressure, are key aspects for reducing the incidence of PPHN. All 12 risk factors included in this study can be considered as relevant risk factors for PPHN, and attention should be paid to newborns with multiple risk factors in clinical practice to provide better awareness for the early diagnosis of PPHN. The risk that cesarean section delivery poses for developing PPHN should be considered when selecting the mode of delivery. Pregnant women should also try to avoid or reduce the use of steroids and SSRI drugs during pregnancy.

## Data Availability Statement

The original contributions presented in the study are included in the article/supplementary material, further inquiries can be directed to the corresponding author.

## Author Contributions

RZ conceptualized and designed the study, drafted the initial manuscript, reviewed, and revised the manuscript. Y-NZ and X-YZ designed the data collection instruments, collected data, carried out the initial analyses, reviewed, and revised the manuscript. Y-YC coordinated and supervised data collection and critically reviewed the manuscript for important intellectual content. All authors approved the final manuscript as submitted and agree to be accountable for all aspects of the work.

## Conflict of Interest

The authors declare that the research was conducted in the absence of any commercial or financial relationships that could be construed as a potential conflict of interest.

## Publisher's Note

All claims expressed in this article are solely those of the authors and do not necessarily represent those of their affiliated organizations, or those of the publisher, the editors and the reviewers. Any product that may be evaluated in this article, or claim that may be made by its manufacturer, is not guaranteed or endorsed by the publisher.
